# Uniportal VATS pleural biopsy in the diagnosis of exudative pleural effusion: awake or intubated?

**DOI:** 10.1186/s13019-021-01461-7

**Published:** 2021-04-20

**Authors:** Mertol Gokce, Bulent Altinsoy, Ozcan Piskin, Burak Bahadir

**Affiliations:** 1 Department of Thoracic Surgery, Bulent Ecevit University Faculty of Medicine, Zonguldak, Turkey; 2 Department of Pulmonary Medicine, Bulent Ecevit University Faculty of Medicine, Zonguldak, Turkey; 3 Department of Anesthesiology and Reanimation, Bulent Ecevit University Faculty of Medicine, Zonguldak, Turkey; 4 Department of Pathology, Bulent Ecevit University Faculty of Medicine, Zonguldak, Turkey

**Keywords:** Awake, Local anesthesia, Pleural effusion, Video-assisted thoracoscopic surgery

## Abstract

**Background:**

The aim of this study is to compare the diagnostic efficacy and safety of video-assisted thoracoscopic surgery (VATS) with awake VATS (AVATS) pleural biopsy in undiagnosed exudative pleural effusions.

**Methods:**

The diagnostic efficacy of pleural biopsy by uniportal VATS under general anesthesia or AVATS under local anesthesia and sedation performed by the same surgeon in patients with undiagnosed exudative pleural effusion between 2007 and 2020 were retrospectively evaluated. Test sensitivity, specificity, positive predictive value and negative predictive value were compared as well as age, gender, comorbidities, procedure safety, additional pleural-based interventions, duration time of operation and length of hospital stay.

**Results:**

Of 154 patients with undiagnosed exudative pleural effusion, 113 (73.37%) underwent pleural biopsy and drainage with VATS, while 41 (26.62%) underwent AVATS pleural biopsy. Sensitivity, specificity, positive predictive value and negative predictive value were 92, 100, 100, and 85.71% for VATS, and 83.3, 100, 100, and 78.9% for AVATS, respectively. There was no significant difference in diagnostic test performance between the groups, (*p* = 0.219). There was no difference in the rate of complications [15 VATS (13.3) versus 4 AVATS (9.8%), *p* = 0.557]. Considering additional pleural-based interventions, while pleural decortication was performed in 13 (11.5%) cases in the VATS group, no pleural decortication was performed in AVATS group, (*p* = 0.021). AVATS group was associated with shorter duration time of operation than VATS (22.17 + 6.57 min. Versus 51.93 + 8.85 min., *p* < 0.001). Length of hospital stay was relatively shorter in AVATS but this was not statistically significant different (*p* = 0.063).

**Conclusions:**

Our study revealed that uniportal AVATS pleural biopsy has a similar diagnostic efficacy and safety profile with VATS in the diagnosis and treatment of patients with undiagnosed pleural effusion who have a high risk of general anesthesia due to advanced age and comorbidities. Accordingly, uniportal AVATS pleural biopsy may be considered in the diagnosis and treatment of all exudative undiagnosed pleural effusions.

## Background

Pleural effusions may develop in many different conditions and the most common causes in developed countries are congestive heart failure, pneumonia and malignancy, respectively [1]. It is estimated that 1.5 million patients suffer from pleural effusion per year [2]. To determine the etiology of pleural fluid, firstly, biochemical, cytological and microbiological analysis of the fluid is required by thoracentesis; however, this procedure allows diagnosis in only about 75–60% of cases [3]. The second step diagnostic procedure in negative pleural effusions of unknown causes is conventional ‘blind’ pleural biopsy, i.e. non-image guided pleural biopsy. This procedure is inexpensive, easy to apply and still used in many institutions. The use of ‘blind’ pleural biopsy increases the diagnostic efficiency of malignant pleural fluid cytology by 7–27% [3]. However, 20–40% of patients with exudative pleural effusion cannot be diagnosed by recurrent thoracentesis and ‘blind’ pleural biopsy [3, 4]. Image guided (computed tomography or ultrasound) pleural biopsy has been shown to have a diagnostic efficiency of 76–85% in undiagnosed exudative pleural effusions [5].

The etiology of exudative effusions varies by population, and the most undiagnosed exudative effusions are associated with malignancy or tuberculosis [2, 6]. Pleural effusion has been reported in approximately 15% of patients who died due to malignancy [7]. Given these reasons, it is very important to diagnose exudative effusions. The definitive diagnosis is based on targeted biopsy of the pleura by two methods: awake thoracic surgery and video-assisted thoracoscopic surgery (VATS) [2–4]. Awake thoracic surgery was first performed in the early 1900s by Swedish doctor Hans Christian Jacobaeus using a cystoscope in the collapse treatment of pulmonary tuberculosis [8, 9]. Although awake thoracoscopy (AT) is the most often used term, it is also named in many different terms such as local anesthetic thoracoscopy, non-intubated thoracoscopy, medical thoracoscopy and etc. AT can be performed by a pulmonologist or surgeon in an endoscopy room, a clean bronchoscopy unit or an operating room under conscious sedation and local anesthesia [10]. In this procedure, the patient is placed in the lateral decubitus position and a flexible disposable trocar is used. Pleural biopsy and drainage are performed using a semi-rigid scope [2, 4, 9, 10]. VATS is performed by a thoracic surgeon in an operating room under general anesthesia and requires one lung ventilation [2, 4, 11]. Pleural biopsy and drainage are performed using a rigid scope. Both techniques allow visualization of the pleura and targeted biopsy. Similar results have been reported in the literature with both techniques in the diagnosis of undiagnosed pleural effusions, particularly of malignant pleural disease [2–4]. AT provides an alternative diagnostic strategy for patients with severe comorbidities that are not suitable for general anesthesia or one lung ventilation [2, 3, 10]. Likewise, awake VATS (AVATS) pleural biopsies are successfully performed under local anesthesia and sedation in awake patients with a high risk of general anesthesia and/or not suitable for one lung ventilation [11–15].

This is a study performed by the same surgeon, comparing the efficacy and safety of uniportal AVATS versus VATS pleural biopsy for undiagnosed exudative pleural effusions as well as the duration time of operation and length of hospital stay.

## Methods

**Fig. 1:**
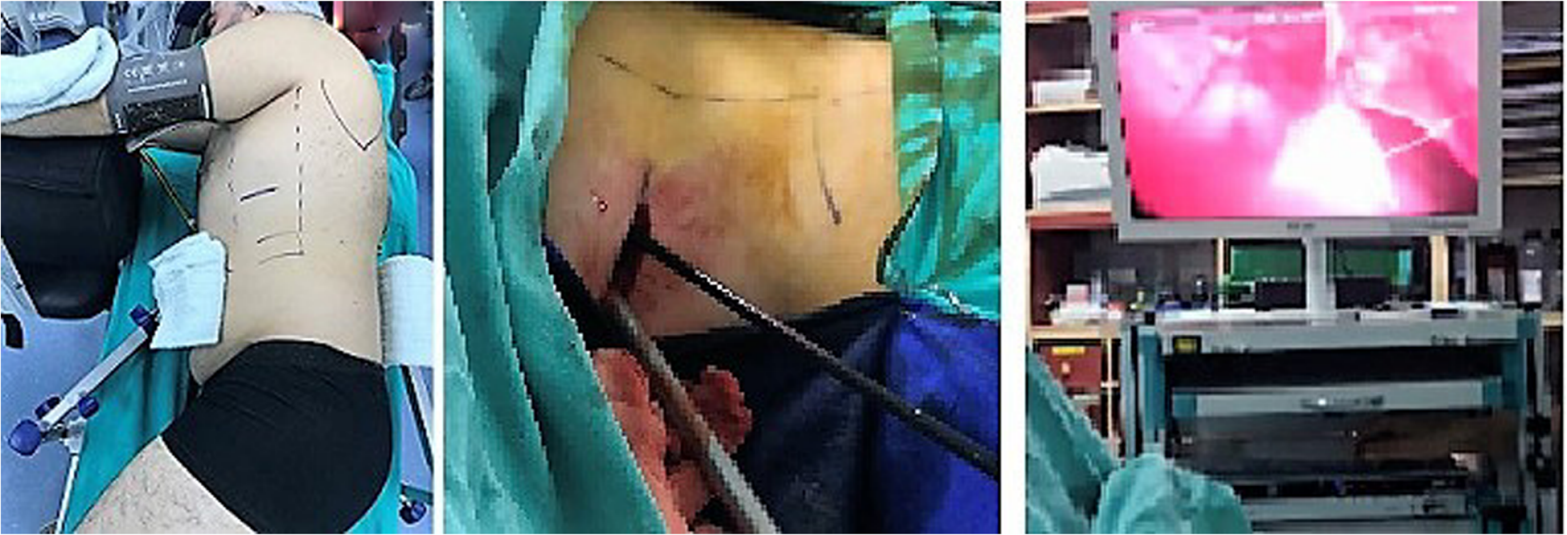
Lateral decubitus position in uniportal video-assisted thoracoscopic surgery (left), rigid video-thoracoscope through a single skin incision (middle) and pleural biopsy with endograsper (right)

**Fig. 2:**
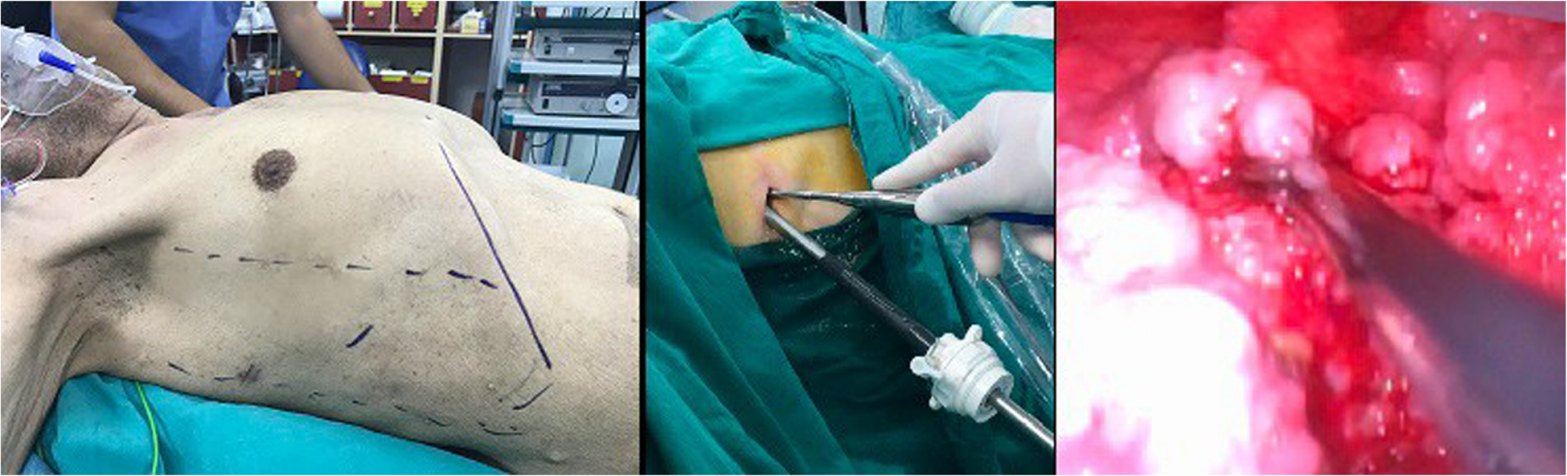
Supine position in uniportal awake video-assisted thoracoscopic surgery (left), rigid video-thoracoscope within the trocar through a single skin incision (middle) and pleural biopsy with endograsper (right)

Patients with exudative pleural effusions referred to the Thoracic Surgery Clinic at Bülent Ecevit University Medical Faculty Hospital who underwent diagnostic uniportal AVATS or VATS pleural biopsy between October 2007 and February 2020 were included in the study. Thoracic surgery and hospital records were reviewed. All procedures were performed in the operation room by the same thoracic surgeon and a 5 mm diameter 30^0^ angled rigid video-thoracoscope (Karl Storz 26,046 BA) was used through the trocar. The pleural space in one hemithorax was entered using a 10 mm single thoracoport through a single skin incision of approximately 20 mm at the junction of the midaxillary line and the fifth or sixth intercostal space. The patients who underwent uniportal VATS pleural biopsy were given lateral decubitus position with the affected hemithorax on top and a small sub-axillary gel roller below the underlying hemithorax was placed (Fig. 1). Uniportal VATS pleural biopsies were performed under general anesthesia and one lung ventilation. The patients who underwent uniportal AVATS pleural biopsy were placed in a supine position on the operating table, and a small gel roller under the operational side was placed (Fig. 2). In uniportal AVATS pleural biopsies, sedation was performed by an anesthesiologist using opioid, benzodiazepine or propofol combinations. Local anesthesia was performed by the surgeon applying lidocaine to the trocar entry. There was no need for conversion to general anesthesia in AVATS patients. At the end of the operation, all patients were extubated and transferred to their beds.

Pathology reports of all pleural biopsies were reviewed. Non-specific biopsy results (acute or chronic inflammation, reactive mesothelial cells, pleural fibrosis, organizing fibrinous pleuritis or normal pleura), samples with malignancy or suspicious for malignancy, insufficient samples and samples reported as granulomatous inflammation were compared with clinical and pathologic outcomes at 12 months after the index procedure. To confirm outcomes, hospital databases were reviewed and patient outcomes that could not be verified for up to 12 months were considered to lost to follow-up.

Test sensitivity, specificity, positive predictive value and negative predictive value were compared. The safety of the procedure was assessed according to the complications previously described in the literature [2–4, 6, 11]. The interval between anesthesia induction and the last skin suture was considered as the duration time of operation. The length of hospital stay was calculated taking into account the time from the day patients were hospitalized to the day they were discharged.

SPSS 19.0 was used for statistical analysis. Descriptive statistics for continious variables are given with median, minimum and maximum values, where categorical variables are given with frequency and percent. Shapiro Wilk test was used for test of normality. Mann Whitney U test was used for two independent group comparisons for non-normal distributed variables. Yates Chi-Square and Fisher Exact Chi-Square tests were used for comparison of categorical variables among groups. For all statistical comparisons with a *p* value below 0.05 was assumed as statistically significant.

This study was approved by the University Research Ethics Comittee (10.06.2020, version no,2020/12–1).

## Results

The results of uniportal AVATS and VATS pleural biopsies in patients with undiagnosed exudative pleural effusion were analyzed retrospectively. A total of 154 patients underwent surgical pleural biopsy. Uniportal VATS (n: 113) and AVATS (n: 41) were performed. The baseline characteristics of the cases are given in Table 1.

**Table 1:** Basic characteristics of cases

Characteristics	Uniportal VATS	Uniportal AVATS	***p*** Value
**Pleural procedures, n**	113	41	
**Age (years): mean** ± **SD**	53.73 ± 18.10	76.17 ± 12.67	< 0.001*
**Male**	80 (70.8%)	35 (85.4%)	0.104
**CCI: mean** ± **SD**	2.30 ± 2.09	5.24 ± 2.27	< 0.001*
No	58 (51.3%)	2 (4.9%)	
Yes	55 (48.7%)	39 (95.1%)	
^**a**^**FEV**_**1**_**%: mean** ± **SD**	71.63 ± 17.77	59.25 ± 15.09	0.007*

*VATS* Video-assisted thoracoscopic surgery, *AVATS* Awake video-assisted thoracoscopic surgery, *CCI* Charlson Comorbidity Index, *FEV1* forced expiratory volume of air in 1 s

^a^ Data are available for 8 AVATS patients and 36 VATS patients

Patients in AVATS group were significantly older than those in VATS group (*p* < 0.001). There was no difference in terms of gender. FEV_1_% mean value was significantly lower in AVATS group than in VATS group, (*p* = 0.007). However, FEV_1_% was available for only 8 AVATS patients and 36 VATS patients, most patients were with incompatible spirometry or patients without spirometry.

Comorbidities of cases in both groups, if any, were also recorded and scored according to Charlson Comorbidity Index (CCI) [16]. There were no comorbidities in 2 patients (4.9%) in AVATS group and in 58 patients (51.3%) in VATS group. Mean value of CCI in AVATS group (5.24 ± 2.27) was significantly higher than VATS group (2.30 ± 2.09), (*p* < 0.001).

**Fig. 3:**
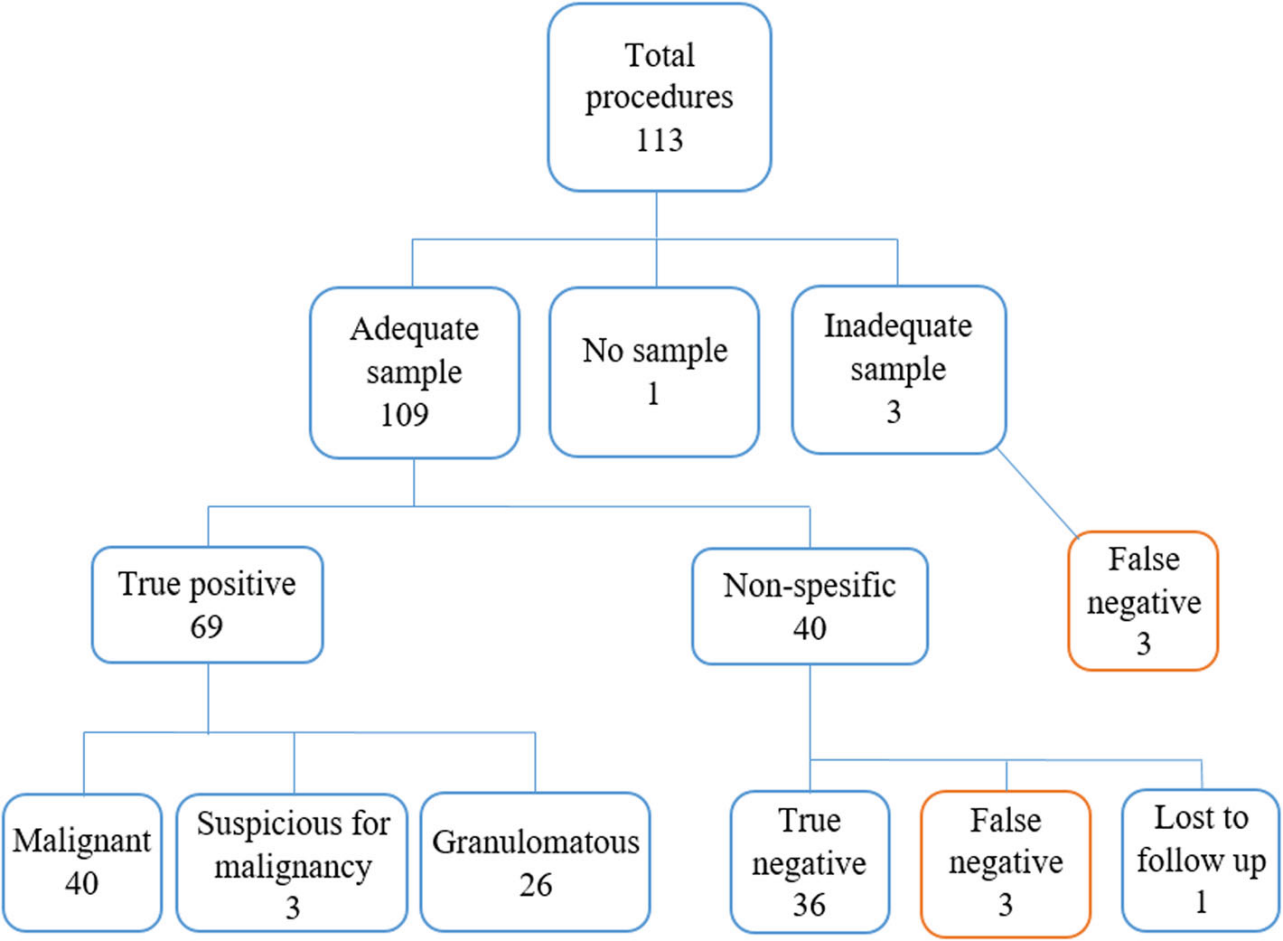
Diagnostic outcome of pleural biopsies obtained by video-assisted thoracoscopic surgery

**Fig. 4:**
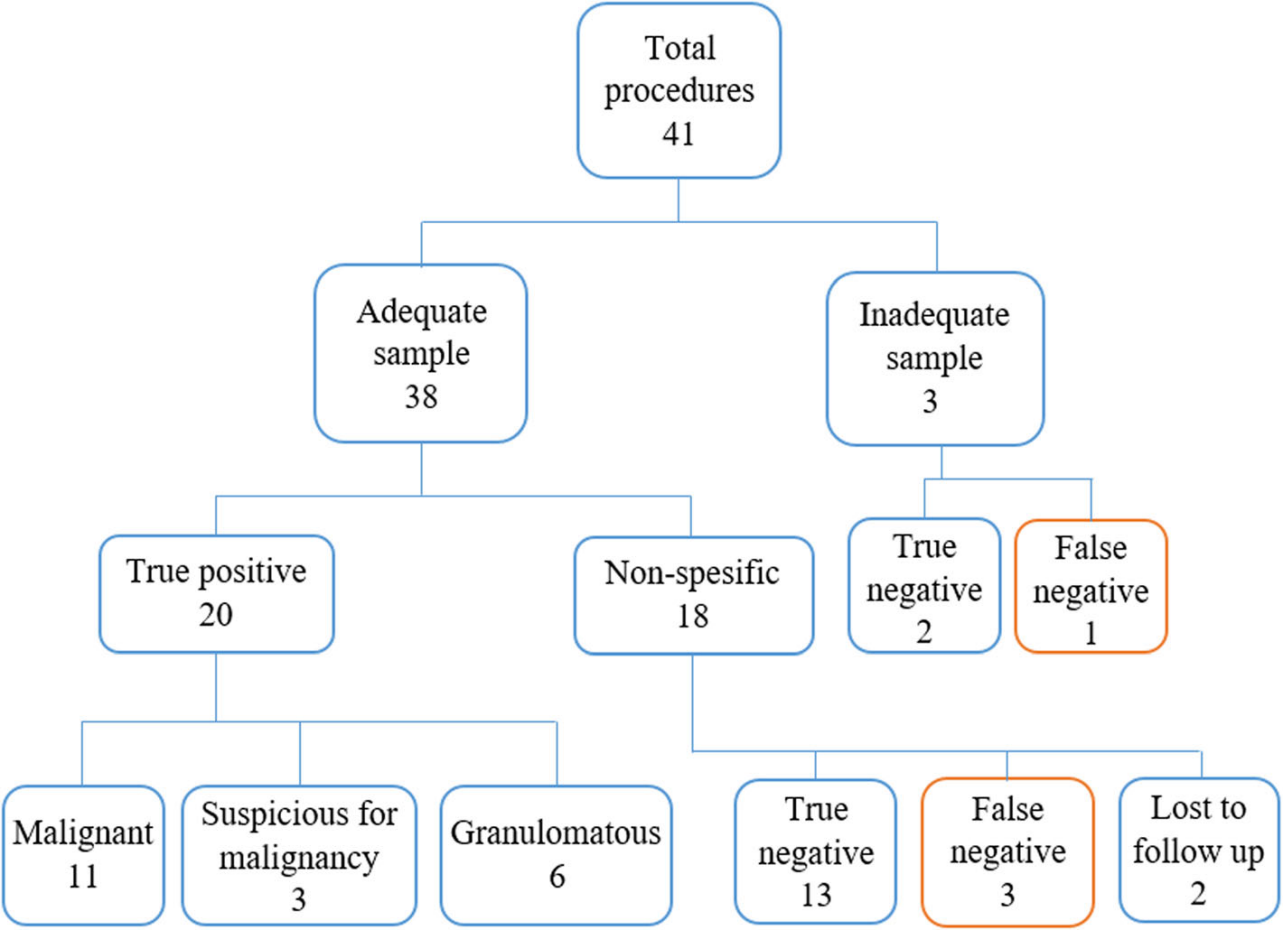
Diagnostic outcome of pleural biopsies obtained by awake video-assisted thoracoscopic surgery

Adequate biopsy samples for pathologic examination were taken in 109 (96.46%) of 113 cases in VATS group and in 38 (92.68%) of 41 patients in AVATS group. In VATS group, 43 cases (38.1%) were malignant, 3 (2.65%) were suspicious for malignancy, 26 (23%) were granulomatous inflammation and 44 (38.9%) were nonspecific (Fig. 3). Biopsy samples were considered insufficient in 3 cases and biopsy sample was not obtained in 1 case. Repeat biopsy results in 3 of these 4 cases were reported as malignant. In AVATS group, 11 cases (26.8%) were malignant, 3 (7.5%) were suspicious for malignancy, 6 (14.6%) were granulomatous inflammation and 21 (51.2%) were nonspecific (Fig. 4). Biopsy specimens were considered insufficient in 3 cases. Repeat biopsy results were true negative in 2 cases and false negative in 1 case. Clinical and pathological follow-up for up to 12 months for non-specific, suspicious for malignancy and insufficient pleural biopsy samples is summarized in Table 2. The most common malignancy in both groups was adenocarcinoma (44.9%) (primary or metastatic), followed by mesothelioma (4.49%) and neuroendocrine cell tumor (4.49%).

**Table 2:** Outcome of suspicious for malignancy, nonspecific and inadequate pleural biopsy results at 12 months

Outcomes	Uniportal VATS (***n*** = 46)	Uniportal AVATS (***n*** = 24)
Suspicious for Malignancy	3	3
Remained suspicious for malignancy	1/3 (33.3%)	2/3 (66.6%)
Confirmed malignant	2/3 (66.6%)	1/3 (33.3%)
No longer suspicious for malignancy	0/3 (0%)	0/3 (0%)
Nonspecific	40	18
Benign	36/40 (90%)	13/18 (72.2%)
Malignant diagnosis	3/40 (7.5%)	3/18 (16.6%)
Suspicious for Malignancy	0/40 (0%)	0/18 (0%)
Lost to follow-up	1/40 (2.5%)	2/18 (11%)
Inadequate sampling	3	3
Malignant diagnosis	3/3 (100%)	1/3 (33.3%)

*VATS* Video-assisted thoracoscopic surgery, *AVATS* Awake video-assisted thoracoscopic surgery

The results used for sensitivity and specificity analysis of all pleural biopsy specimens in VATS and AVATS groups are summarized in Figs. 3 and 4, respectively. Sensitivity, specificity, positive predictive value and negative predictive value were 92, 100, 100, and 85.71% in VATS group, respectively. Sensitivity, specificity, positive predictive value and negative predictive value were 83.3, 100, 100, and 78.9% in AVATS group, respectively. There was no significant difference in diagnostic test performance between the groups, (*p* = 0.219).

Complications were observed in 15 cases (13.3%) in VATS group; loculated effusion in 5 cases (4.42%), prolonged air leakage and expansion defect in 3 (2.65%), self-limiting subcutaneous emphysema in 2 (1.76%), empyema and bronchopleural fistula in 2 (1.76%), cardiac arrhythmia in 2 (1.76%), and long pleural drainage in 1 (0.88%). Complications were observed in 4 cases (9.8%) in AVATS group; expansion defect in 2 cases (4.87%) and loculated effusion in 2 cases (4.87%). There was no statistically significant difference in complication rates between the groups (*p* = 0.557). The type and frequency of complications are shown in Table 3. There was no mortality associated with the procedures.

**Table 3:** Type and frequency of complications

Complications	VATS (*n* = 113)	AVATS (*n* = 41)
Death within 30 days	0	0
Loculated effusion	5	2
Prolonged air leakage and expansion defect	3	2
Self-limiting subcutaneous emphysema	2	0
Empyema and broncho-pleural fistula	2	0
Cardiac arrhythmia	2	0
Long pleural drainage	1	0

*VATS* Video-assisted thoracoscopic surgery, *AVATS* Awake video-assisted thoracoscopic surgery

Duration of operation was 51.93 ± 8.85 min (40–80 min.) in VATS group and 22.17 ± 6.57 min (17–60 min.) in AVATS group. The difference between groups was statistically significant, (*p* < 0.001).

The average length of hospital stay was 2.26 days (SD ± 0.70) in AVATS group and 2.67 days (SD ± 1.56) in VATS group. There was a relatively shorter hospital stay in AVATS group, but there was no statistically significant difference betweens groups (*p* = 0.063).

Talc pleurodesis was performed in 16 patients in VATS group and in only 1 patient in AVATS group. There was a significant difference between the groups, (*p* = 0.043).

While taking pleural biopsy pleural decortication was performed as an additional diagnostic and treatment procedure in 13 patients with pleural thickening in VATS group. No pleural decortication was performed in AVATS group. There was a significant difference between the groups, (*p* = 0.021).

## Discussion

In this study, we have revealed that diagnostic efficiency of uniportal VATS and AVATS pleural biopsies are similar in patients with undiagnosed exudative pleural effusion. There were few complications in both groups and there was no statistically significant difference in complication rates between the groups. This was compatible with previous studies demonstrating AT safety in patients at high risk of complications due to advanced age, comorbidity and performance status [4, 10, 12–15, 17–19]. Patients with pleural effusion are the most suitable candidates for AVATS, due to the frequent presence of medical comorbidities which imply additional general anesthesia related risk [12, 20, 21]. Our findings indicated that calculating the CCI scores of patients scheduled for targeted pleural biopsy may guide the selection of the surgical method (VATS or AVATS).

In our study, pleural biopsy with AVATS was performed only under local anesthesia and sedation. Many major thoracic operations have been successfully performed with AVATS, but most were performed with thoracic epidural anesthesia or paravertebral blockage [12, 21–23]. Thoracic epidural anesthesia is not only difficult to apply, but is also associated with a higher risk of dural puncture and spinal cord injury than the lumbar region [24, 25]. In addition, thoracic epidural anesthesia is not appropriate for all patients and is contraindicated in patients with previous spinal surgery, blood clotting disorders, anticoagulant therapy, and local infections.

In most studies with AT pleural biopsy, the patient position is almost the same as VATS; i.e. the patient is placed on the operating table in the lateral decubitus position with the affected hemithorax on top [2, 4, 9, 10]. Alrawi et al. (2002) performed AVATS pleural biopsy and drainage in a supine position under local anesthesia and sedation in patients with pleural effusion [20]. Mean duration of operation time was 62 min. The average chest tube duration was 6 days. No mortality was observed and morbidity was 10% (2 patients intubated). The patients were followed for 12 months and no recurrence or sequelae developed. Klijian et al. (2014) analyzed 293 patients undergoing AVATS including wedge resections, lobectomies, decortications, pleural biopsies, pleurodesis, bullectomies, and pericardial windows under local anesthesia with sedation. The patients were given supine position with a small gel roller under the operation side [12]. Most of the patients had comorbidities (111 diabetes mellitus, 114 chronic obstructive pulmonary disease, 30 atrial fibrillation, and 118 hypertension). Overall, there were only 14 complications, but no mortality. The average chest tube duration was 1.2 days. Length of hospital stay was 2.5 days for 68 decortications and 1 day for 33 pleural biopsies. Similarly, Katlic et al. (2015) performed AVATS in a supine position under local anesthesia and sedation in 96 patients aged between 80 and 104 years [26]. Procedures included pleural biopsy/effusion drainage with or without talc, empyema drainage, hemothorax, pericardial window, lung biopsy, chylothorax, and pneumothorax. Local anesthesia and sedation were recommended for patients with large unilateral pleural effusion and empyema. Mean duration of operation time for pleural effusion and empyema was 24 min. No patient required intraoperative intubation or epidural or nerve block analgesia. Three patients died after complications (3.1% morbidity and 3.1% mortality). As concluded from studies by Alrawi et al. (2002), Klijian et al. (2014) and Katlic et al. (2015), it can be argued that in parallel with the AVATS surgical experience and the development of VATS devices, both the operation time and duration of hospital stay have significantly shortened and the variety of operations has increased [12, 20, 26]. In all these studies, the authors also stated that intubation can be easily performed in the supine position when urgent intubation is required, but it is difficult in the lateral decubitus position [12, 20, 26]. The lateral decubitus position has some effects on ventilation and perfusion. The gravity dependent distribution of flow is maintained, with a roughly 10% shift of cardiac output to the dependent lung [27, 28]. Similar to pulmonary perfusion, gravitational forces affect the distribution of ventilation across the lung. In addition, hypoxic pulmonary vasoconstriction actively increases vascular resistance in the lung without ventilation, which causes a gradual decrease in the shunt fraction [28]. In our study, supine position was preferred in AVATS patients to provide easy intubation when urgent intubation is required. Moreover, supine position prevents 10% shunt in the cardiac output that may occur in the lateral decubitis position, this may be particularly important in patients with high CCI.

Duration time of operation in the current study was also statistically significantly shorter in AVATS group (22.17 ± 6.57 min.) than in VATS group. This was clearly due to duration of intubation for the one lung ventilation and lateral decubitus position in VATS group. Chest tube retention was shorter in uniportal AVATS patients, resulting in relatively shorter hospital stay (2.26 days ± 0.70) but there was no statistically significant difference between the groups. This was probably due to the absence of outpatients in our study. All patients were hospitalized at least one day before surgery and were discharged at least one day after surgery. Since uniportal AVATS patients were older and had high CCI scores, we did not consider to discharge on the same day due to risk of developing complications. Contrary to our study, in some studies with AT, the length of hospital stay was found to be significantly shorter. However, in these studies, patients with long hospital stay were excluded from the study [3, 4, 19]. Due to the health policies of countries, differences in billing can be in a wide variety. The factors that determine the cost analysis in these procedures are the medication, consumables used, and hospital stay. In a study conducted in Canada, the average approximate cost of AT pleural biopsy was 2815 Canadian dollars, while VATS pleural biopsy was 7962 Canadian dollars [4]. In a multi-center randomized study conducted in our country, the average cost of AVATS pleural biopsy was about $87, while VATS pleural biopsy was $134 [29].

Subsequent to pleural biopsy, we applied talc pleurodesis to patients considered to have malignancy. When the lung was fully re-expanded, 2–4 g talc was blown intrapleurally under direct vision and a chest tube was placed [30]. Talc pleurodesis was performed in significantly more VATS cases as compared with AVATS cases. The answer to the question of why we applied less pleurodesis to AVATS is that cases in which the lung is not fully expanded and cases not considered as malignancy are more in this group. In case of extensive adhesiolysis and pleural thickening while performing pleural biopsy with VATS, pleurectomy and decortication process should be added to the operation [18, 19]. This situation has not been clearly stated in publications on AT [3, 4]. We performed pleural decortication in 13 cases in VATS group, since VATS allows pleural decortication by providing wider vision and better surgical maneuver under one lung ventilation [31, 32].

In our study, the diagnostic efficacy of both uniportal AVATS and uniportal VATS for undiagnosed exudative pleural effusion was similar to recent studies [4, 17, 18, 20]. All cases with nonspecific biopsy results in our study were followed-up clinically and pathologically for up to 12 months. In previous studies, approximately 45% of pleural biopsies were diagnosed with nonspecific pleuritis [33]. In the follow-up of these patients, most common malignancy diagnosed was mesothelioma (5–25%) [31, 33, 34]. Most malignancies were detected within the first 1 year after biopsy [33]. Another study showed that 12.5% of nonspecific biopsy specimens in AT group and 17.4% in the VATS group were suspected for malignancy at follow-up [4]. In our study, 16.6% of nonspecific biopsy specimens in the uniportal AVATS group and 7.5% in the uniportal VATS group proved to be malignant or suspicious for malignancy on follow-up. This finding clearly emphasizes the importance of clinical follow-up of patients with non-specific pleural biopsy results.

Local awake procedures provide lower costs, shorter hospital stay, shorter anesthesia time, and shorter operation time compared to general anesthesia patients [12, 35]. Other advantages include increased ventilation, less respiratory complications, and shorter recovery time. Finally, local anesthesia is not traumatic for the immune system which allows for faster recovery [36]. The biggest advantage of VATS is that it allows for a wide view of a hemithorax due to one lung ventilation, thereby providing an opportunity for biopsy on suspicious pleural lesions and nodules on the surface of the lung with direct vision [31, 32]. In addition, when there is a need for decortication in cases accompanied by pleural thickening, total or partial decortication can be performed with uniportal VATS. Unfortunately, it is very difficult to do this in cases where decortication is required in AT. In small pleural effusions or in the absence of pleural effusions, iatrogenic pneumothorax may be created by a qualified operator and a thoracoscope is inserted, and the pleural cavity can be imaged and biopsied in AT [3]. However, pleural biopsy can be more easily performed with VATS with one lung ventilation. In our experience, uniportal AVATS is neither effective nor safe for small pleural effusions. Because it is difficult to place a port on the hemithorax in these patients and there is a risk of parenchymal injury.

### Study limitations

Our study was retrospective and therefore biased. We selected only patients with undiagnosed pleural effusion by uniportal AVATS or VATS pleural biopsy to ensure that both patient populations were as similar as possible. The study is based on a single thoracic surgery clinic and therefore may not apply to those with different patient populations. Another bias may be that we mostly performed uniportal AVATS in cases with a CCI score of 5 or higher. In addition, data collection was limited to patient records. One patient in uniportal VATS and two patients in uniportal AVATS group were lost to follow-up; however, this is not expected to significantly affect the results of the study.

## Conclusions

Uniportal AVATS and VATS pleural biopsy have a similar diagnostic efficacy and safety profile in the evaluation of undiagnosed exudative pleural effusions. We have demonstrated that uniportal AVATS pleural biopsy and drainage can be used as an effective and safe tool in the diagnosis and treatment of patients with undiagnosed pleural effusion who have a high risk of general anesthesia due to advanced age and comorbidities. As a result, we consider that uniportal AVATS pleural biopsy can be routinely used in the diagnosis and treatment of all exudative undiagnosed pleural effusions.

## Data Availability

The datasets used and /or analysed during the current study are available from the corresponding author on reasonable request.

## Abbreviations

VATS: Video-assisted thoracoscopic surgery
AT: Awake thoracoscopy
AVATS: Awake video-assisted thoracoscopic surgery
FEV_1_: Forced expiratory volume of air in 1 s
CCI: Charlson Comorbidity Index
